# Non-invasive assessment of intracranial wall shear stress using high-resolution magnetic resonance imaging in combination with computational fluid dynamics technique

**DOI:** 10.1016/j.fmre.2021.09.019

**Published:** 2021-11-27

**Authors:** Yuwen Chen, Jia Liu, Mingli Li, Yannan Yu, Zhengzheng Yan, Wenshin Shiu, Bokai Wu, Zaiheng Cheng, Yao Meng, Yang Liu, Weizhuang Yuan, Zongmuyu Zhang, Weihai Xu

**Affiliations:** a Department of Neurology, State Key Laboratory of Complex Severe and Rare Diseases, Peking Union Medical College Hospital, Chinese Academy of Medical Sciences and Peking Union Medical College, 1 Shuaifuyuan, Beijing 100005, China; b Department of Cardiology, Second Affiliated Hospital of Zhejiang University School of Medicine, Hangzhou, Zhejiang 310009, China; c Shenzhen Institutes of Advanced Technology, Chinese Academy of Sciences, Shenzhen 518055, China; d Shenzhen Key Laboratory for Exascale Engineering and Scientific Computing, Shenzhen 518055, China; e Department of Radiology, Peking Union Medical College Hospital, Chinese Academy of Medical Sciences, 1 Shuaifuyuan, Beijing 100005, China; f Department of Radiology, Stanford University, California 94305, United States; g Department of Neurology, Saarland University, Kirrberger Straße 66421, Germany

**Keywords:** Intracranial atherosclerosis, Wall shear stress, Atherosclerotic plaque, High-resolution magnetic resonance imaging, Computational fluid dynamics

## Abstract

In vivo studies on association between wall shear stress (WSS) and intracranial plaque are deficient. Based on the three-dimensional T1-weighted high-resolution magnetic resonance imaging (3DT1 HR-MRI) data of patients with low-grade stenotic (<50%) atherosclerotic middle cerebral artery (MCA) and subjects with normal MCA, we built a three-dimensional reconstructed WSS model by computational fluid dynamics (CFD) technique. Three-dimensional registration of the CFD model to the HR-MRI was performed with projections based on the resolution and thickness of the images. The relationships between the WSS at each side of the vessel wall and plaque location were analyzed. A total of 94 MCA plaques from 43 patients and 50 normal MCAs were analyzed. In the normal MCAs, WSS was lower at the ventral-inferior wall than at the dorsal-superior wall (proximal segment, *p* < 0.001; middle segment, *p <* 0.001) and lower at the inner wall than at the outer wall of the MCA curve (*p <* 0.001). In atherosclerotic MCAs, similar low WSS regions were observed where plaques developed. The WSS ratio of the ventral-inferior wall to the dorsal-superior wall in atherosclerotic MCAs was lower than that in normal MCAs (*p* = 0.002). The WSS_inner-outer_ ratio in atherosclerotic MCAs was lower than that in normal MCAs (*p* = 0.002). Low WSS was associated with MCA atherosclerosis formation and occurred mainly at the ventral-inferior wall, which was anatomically opposite the orifices of penetrating arteries, and at the inner wall of the MCA curve. Overall, the results were well consistent with the low WSS theory in atherosclerosis formation. The reconstructed WSS model is a promising novel method for assessing an individualized vascular profile once validated by further studies.

## 1 Introduction

Intracranial atherosclerosis is a common cause of stroke, especially in people of Asian, Hispanic, and African descent [1,2]. The pathophysiology of intracranial atherosclerosis remains less well studied compared with that of the peripheral arteries. Conventional cardiovascular risk factors, such as hypertension, hyperlipidemia and smoking, contribute to the pathogenesis of intracranial atherosclerosis. However, they cannot explain why intracranial plaques tend to form in specific regions, such as the internal carotid artery bifurcation and the ventral or inferior walls of the middle cerebral artery (MCA) [3,4]. Studies on coronary artery atherosclerosis have provided clues regarding the role played by local low wall shear stress (WSS) in promoting plaque development and progression. However, in vivo studies on the association between WSS and intracranial plaque are deficient.

Computational fluid dynamics (CFD), a well-established engineering technique, can be used to investigate vascular hemodynamic factors based on computational processing of patient-specific images [5,6]. Time-of-flight (TOF) magnetic resonance angiography (MRA) or computed tomography angiography was used in previous study, in which its usage was limited [7]. Recently, the emerging high-resolution magnetic resonance imaging (HR-MRI) technique has been shown to be capable of optimizing MR images by decreasing slice thickness and in-plane voxel size so that the details of the intracranial vessel wall structure can be assessed accurately. Theoretically, using HR-MRI in combination with the CFD technique would be more precise. In this study, we aimed to evaluate feasibility of WSS based on HR-MRI and analyze the correlations between WSS and MCA plaques.

## 2 Material and methods

### 2.1 Subjects

We retrospectively reviewed our institutional HR-MRI database from March 2015 to January 2018. Patients were recruited if they met the following criteria: (1) presence of atherosclerotic plaques at the M1 segment of the MCA; and (2) < 50% MCA stenosis degree on MRA. Individuals were enrolled as the control group if they fulfilled the following criteria: (1) no history of stroke or transient ischemic stroke; and (2) normal MCA wall on HR-MRI. Participants with poor image quality due to motion artifacts, coexistent ipsilateral internal carotid artery stenosis (>50%), or high-grade MCA stenosis (>50%) were excluded from the study.

The baseline blood pressure prior to the imaging study, peak systolic velocity of the bilateral carotid and vertebral arteries, and the diameter and resistance index (RI) of the bilateral vertebral arteries were obtained through color duplex ultrasonography. The RI of the bilateral internal carotid arteries was calculated manually and was further adjusted based on the RI ratio stratified by age discussed elsewhere [8]. All of the participants or their legal representatives gave their informed consent. This study was approved by the ethics committee at Peking Union Medical College Hospital.

### 2.2 MR protocol

All studies were performed on a 3.0 T scanner (GE Discovery MR750) with an 8-channel head coil. Conventional 3D TOF-MRA was obtained in an axial plane with the following parameters: repetition time (TR) 27 ms, echo time (TE) 6.9 ms, flip angle 20°, field-of-view (FOV) 24 cm × 16 cm, matrix 320 × 256, slice thickness 1.6 mm, and number of excitations (NEX) 1. To identify the atherosclerotic plaques of the M1 segment, 3D CUBE T1-weighted imaging was performed with the following parameters: TR 567 ms, TE 16 ms, FOV 20.4 cm, matrix 320 × 256, slice thickness 0.8 mm, NEX 1, ETL 24. Scan plan: coronal, locs per slab: 128, excitation mode: selective. Phase acceleration 2, zero-filled Fourier transform (ZIP 512, ZIP2) was used to reduce pixel size, and the final display resolution was approximately 0.4 mm × 0.4 mm. A total of 256 coronal slices covering the anterior and posterior circulation (range 9.6 cm) were acquired with a scan time of 5 min. The fat suppression technique was used to reduce fat signals from the scalp.

### 2.3 Image analysis

**Fig 1:**
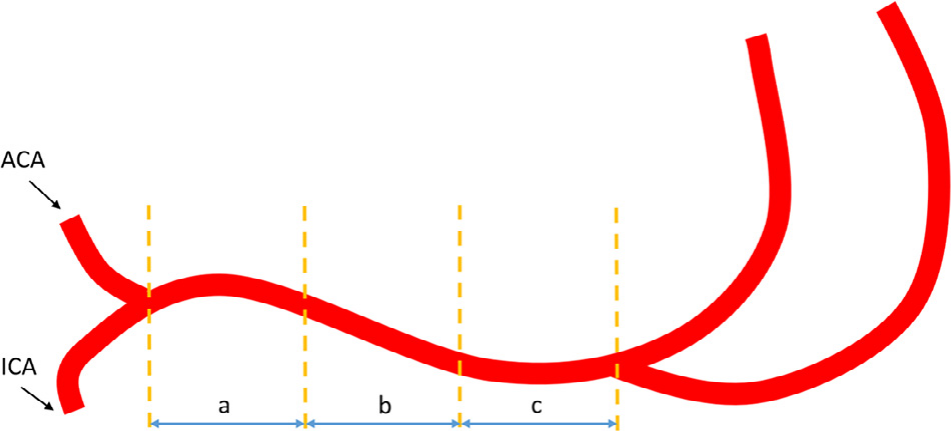
**Three segments of the horizontal part of the middle cerebral artery.** (a). Proximal segment: from the bifurcation of the internal carotid artery and anterior cerebral artery to the first trisection point; (b). Middle segment: from the first trisection point to the second; (c). Distal segment: from the second trisection point to the distal bifurcation of the middle cerebral artery.

**Fig 2:**
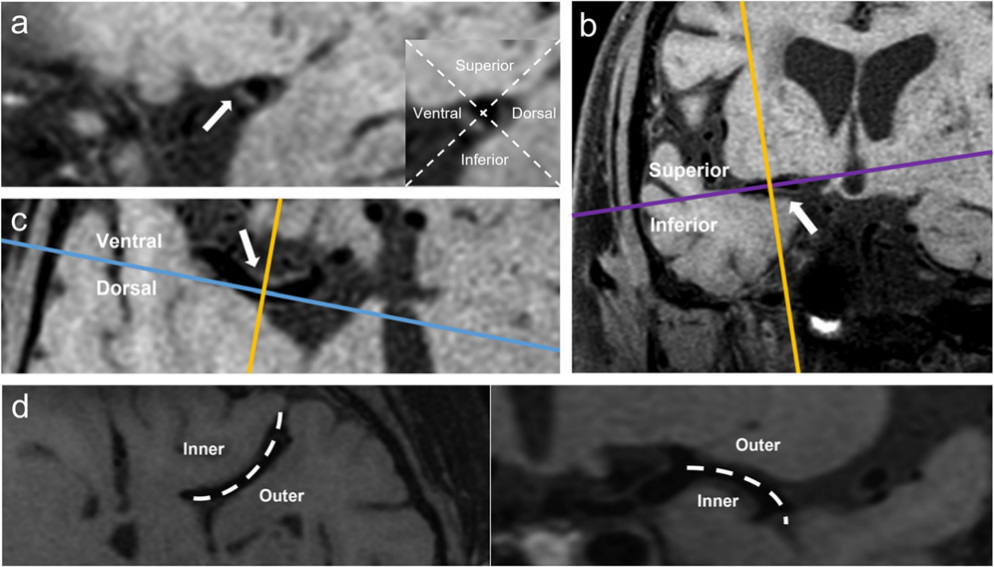
**Vessel walls and middle cerebral artery (MCA) Illustration of a plaque involving the ventral-inferior walls of MCA and a curved M1 segment.** (a). Sagittal plane (represented by the yellow line); (b). Coronal plane (represented by blue line); (c). Axial plane (represented by the purple line); the blue line separates the ventral and dorsal walls on the axial plane; the purple line separates the superior and inferior walls on the coronal plane; (d). Axial and coronal planes; the inner and outer walls were determined based on the geometry of the MCA curve.

It is known that the branch arteries and artery geometry may affect the distribution of the WSS [9,10]. In the MCAs, the perforating arteries of the M1 segment are usually divided into medial, intermediate, and lateral groups, each of which is responsible for the blood supply in various territories [11]; therefore, we divided the M1 segment into 3 equal parts (proximal segment, middle segment, and distal segment) based on the location of the trisection points from the beginning of the MCA to the first branch point of the MCA (Fig. 1). The vessel walls were further classified into the superior, inferior, ventral, and dorsal walls according to the cross-section of the MCA viewed on the sagittal plane of the 3D T1-weighted HR-MRI image [3] (Fig. 2). Our previous study found that curved M1 segments were predominant regardless of the existence of atherosclerotic plaques [12]. Based on the MCA geometry shown in the axial and coronal views on HR-MRI, the 4 vessel walls of the middle segment of the MCA were classified into the inner and outer walls of the curves [12] (Fig. 2).

On HR-MRI, an MCA plaque was identified if there was eccentric wall thickening, of which the thinnest part was less than 50% of the thickest point by visual inspection [13]. All plaques were classified into ventral, dorsal, superior, and inferior groups based on the wall on which their centrals were located.

### 2.4 CFD Modeling

#### 2.4.1 3D computational domains

The MRA data of each patient were exported in the standard Digital Imaging and Communication in Medicine (DICOM) format. The cerebral arteries on each MRA image were segmented and reconstructed into a 3D geometry of the vascular surface by Mimics (Materialise NV, Belgium). The reconstructed 3D geometry was examined and manually refined by two neurologists (Yuwen Chen, Yao Meng.). A computational domain (volume mesh) for each subject was generated using ANSYS_ICEM CFD meshing software (ANSYS, Inc., USA). Considering the complexity of the geometry of the cerebral arteries, we employed an unstructured tetrahedral cell for the domain discretization and decreased the mesh size near the vessel wall and at the stenosis, where more precise computation of the hemodynamics was required. The total number of voxels was above 1 million, and the minimum volume of the voxels required to capture the microscale dynamics of the blood flow was approximately 1.0 × 10^−8^ cm^3^.

#### 2.4.2 3D model of the blood flow

The blood flow was assumed to be a viscous and incompressible Newtonian fluid. The blood parameters were defined by a constant density $\rho =1.06\;\times 10^{3}\;\mathrm{kg}\cdot m^{-1}$ and a constant dynamic viscosity $\mu =3.5\;\times 10^{-3}\;\mathrm{kg}\cdot m^{-1}\cdot s^{-1}$. We chose the carotid artery diameter $D=6.0\;\times 10^{-3}\;m$ and $v=0.4\;m\cdot s^{-1}$ as the characteristic length and velocity, respectively, of the model. The Reynolds number can thus be calculated as Re=ρvD/μ≈121, which indicates that the flow is laminar. Finally, the governing equation of blood flow can be described by the 3D unsteady incompressible Navier-Stokes equations:

(1.1) $$\frac{\partial v}{\partial t}+\left(v\cdot \nabla \right)v=-\frac{1}{\rho }\nabla p+\frac{\mu }{\rho }\nabla^{2}v+f$$

and the equation of mass conservation can be expressed as:

(1.2) ∇.v=0

where v is the velocity vector, ρ is the density, p is the pressure,μ is the dynamic viscosity, and f is the body force, which is assumed to be equal to 0.

The initial conditions were set to zero for all cases, the details of inlet and outlet boundary conditions were discussed in the next section, and the vessel wall was considered as a no-slip boundary. The solver of the 3D blood flow was based on a finite element method [14] and a domain decomposition-based parallel fully coupled method [15]. The numerical simulations were carried out on the Tianhe 2 supercomputer at the China National Supercomputer Center in Guangzhou. The compute node has a dual six-core Intel Xeon X5650@2.76 GHz processor and 24 GB of memory. A k-way Metis algorithm [16] was applied for the mesh partition in parallel computing, and MPI was used for communication among processor cores. An implicit backward Euler scheme was applied for the transient term, and the time step size was 0.01 s. Relative residual stopping conditions were used for the linear and nonlinear solvers, which were $10^{-4}\;$and $10^{-6},\;$respectively.

#### 2.4.3 Boundary conditions

The patient-specific boundary conditions of the CFD model are given as follows. The inflow rate was calculated from the patient's carotid ultrasonogram at each of the carotid arteries and vertebral arteries by $Q_{in}=V_{in}\cdot A_{in}$, where $Q_{in}$ was the inflow rate in one of the inflow arteries, $V_{in}$ was the mean blood flow velocity, estimated by $V_{in}=\;\frac{1}{2}V_{systole}+\frac{2}{3}V_{diastole}\;$from the ultrasound Doppler signal, and $A_{in}$ was the cross-sectional area, computed by $A_{in}=\;2\cdot \pi \cdot r^{2}$ (r for radius also measured by ultrasonography). The outlet pressure was computed by the integral of the multiplication between the outlet resistance and the outflow rate, $P_{out}=\int R_{out}Q_{out}$ ($Q_{out}$ was calculated by the CFD model; $R_{out}$ was assigned from $R_{total}=P/Q_{in}$; P was the patient's brachial blood pressure measured by the oscillometric method).

#### 2.4.4 3D registration of the CFD model to the T1 HR-MRI images

**Fig 3:**
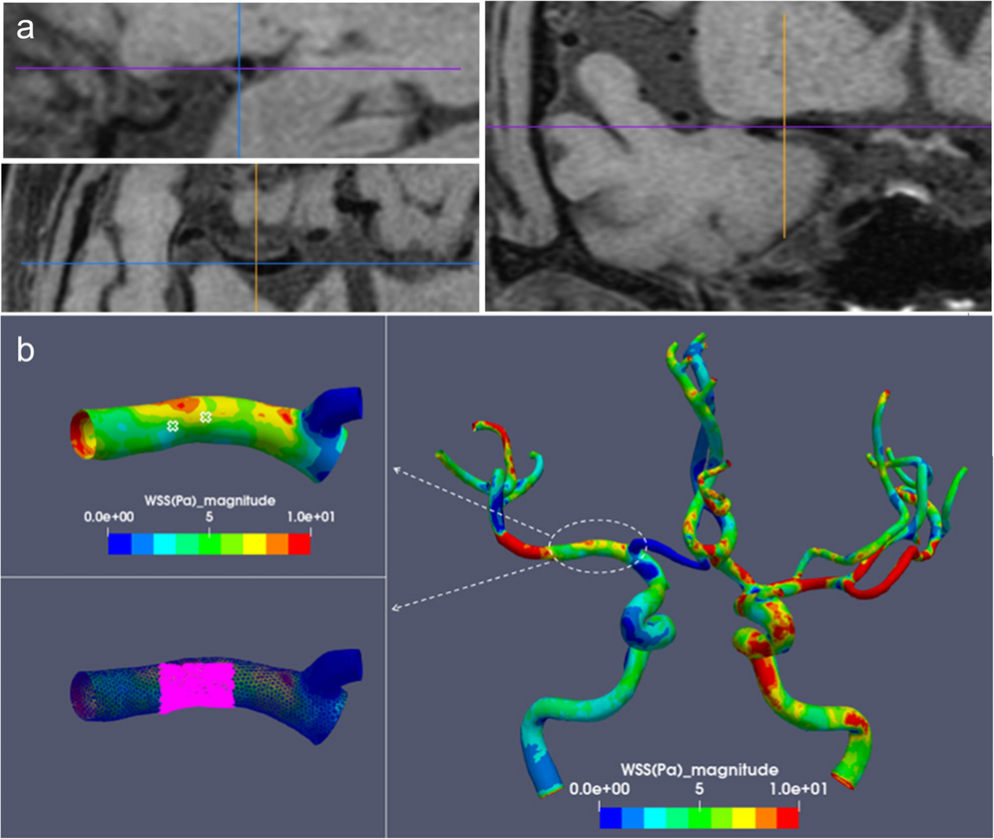
**Plaque localization by a three-dimensional coordinate system and wall shear stress estimation on a three-dimensional reconstructed model.** The plaque was selected on three-dimensional T1-weighted high-resolution magnetic resonance imaging (3D T1 HR-MRI). A coordinate (x, y, z) is shown based on a 3D coordinate system set preconditionally, which corresponds to the coordinate system of the 3D reconstructed wall shear stress (WSS) model. (a). The input coordinates (x, y, z) on 3D T1 HR-MRI are used to localize the selected point on the 3D reconstructed WSS model; each plaque is determined by 2-5 points; (b). The estimated WSS of the plaque-containing ventral and inferior walls; the cross sign indicates plaque location, and the pink spotted area represents the location of the WSS estimation.

The resultant 3D CFD model could be projected to the MRI images according to the resolution and thickness of the images. The coordinate of each element in the 3D CFD model corresponding to the T1 HR-MRI images was calculated by an in-house routine coded in MATLAB (MathWorks, US). Therefore, we could pinpoint the calculated WSS on the raw T1 HR-MRI. ParaView (version 3.98, NTESS LLC, Kitware Inc.) was used to display the 3D reconstructed WSS model. The magnitude of the WSS at the four blood vessel walls within each region of interest was calculated. The mean of the calculated magnitude was used to define the average level of WSS for a particular region (Fig. 3).

#### 2.4.5 Wall shear stress estimation

The WSS was estimated by the following formula:

WSS=σn−(σn·n)n,

where σ is the Cauchy stress tensor.

Since the WSS was not directly comparable between different artery segments and different individuals, we calculated two WSS ratios to make comparisons among the different segments and subjects: rWSS_VI-DS_ = WSS of the ventral-inferior walls/WSS of the dorsal-superior walls rWSS_inner-outer_ = WSS of the inner wall of the MCA curve/WSS of the outer wall of the MCA curve

### 2.5 Statistical analysis

Quantitative data are expressed as the means ± SDs, and qualitative data are expressed as percentages. For each plaque, the WSS of the plaque-containing walls was compared with that of the normal walls by a paired sample t-test. For each MCA of the control group, comparisons among the WSSs of the four vessel walls and the WSS ratios of the three segments were assessed by one-way ANOVA for continuous variables. Student's t-test was performed to compare the WSS ratios of the plaque and control groups. Fisher's exact test was used for categorical variables. Cohen's κ coefficient and the intraclass correlation coefficient were used to assess interobserver and intraobserver agreement. A Cohen κ coefficient and intraclass correlation coefficient greater than 0.75 were considered excellent. SPSS 23.0 (SPSS Inc, Chicago, IL) was used to perform the statistical analysis. A P-value of <0.05 was considered statistically significant.

## 3 Results

Fifty-seven patients with MCA plaques were enrolled. Four patients with poor image quality, three patients with high-grade stenotic (>50%) intracranial internal carotid arteries, and seven patients with high-grade MCA stenosis (>50%) were excluded. Finally, the HR-MRI data of 43 patients (M1 segment plaques, n=94) were analyzed to estimate the WSS, while 25 individuals with 50 normal MCAs were included as controls. All the participants’ clinical characteristics were recorded (Table 1). The intraobserver and interobserver agreements of the identification of plaque location and the WSS estimations were considered excellent (Table 2).

**Table 1:** **Clinical characteristics of all participants**.

Characteristics	Control Group n = 25 (50 MCAs)	Patient Group n = 43 (94 plaques)	*p*
Age (year, x ± SD)	49.3 ± 16.0	56.4 ± 15.0	0.07
Male (%)	8 (32.0)	34 (79.1)	<0.001
Hypertension (%)	7 (28.0)	27 (62.8)	0.011
Diabetes mellitus (%)	2 (8.0)	14 (32.6)	0.036
Hypercholesterolemia (%)	9 (36.0)	22 (51.2)	0.313
Smoking (%)	5 (20.0)	20 (46.5)	0.038
Cardiovascular disease (%)	4 (4.0)	7 (16.3)	0.242
Stroke history (%)	0 (0)	19 (44.2)	—

**Table 2:** **Intraobserver and interobserver agreement of the identification of plaque location and WSS estimation**.

	Intraobserver agreement (95% CI)	Interobserver agreement (95% CI)
Plaque location	0.751 (0.561-0.941)	0.745 (0.541-0.949)
WSS estimation	0.975 (0.938-0.990)	0.950 (0.922-0.964)

Abbreviation: CI = confidence interval.

The intraobserver and interobserver agreement of plaque location is expressed as the Cohen κ coefficient; WSS estimation is expressed as the intraclass correlation coefficient. A coefficient > 0.75 is considered excellent.

### 3.1 WSS distribution of normal MCAs

In the 50 normal MCAs, the average WSS of the ventral and inferior walls was lower than that of the superior and dorsal walls at both the proximal and middle segments of the MCA (6.53 ± 2.34 Pa vs. 7.79 ± 2.60 Pa at the proximal segment, *p <* 0.001; 6.41 ± 2.73 Pa vs. 8.91 ± 2.76 Pa at the middle segment, *p <* 0.001; Table 3). Similar findings were revealed at the distal segments, but with no statistically significant difference (6.73 ± 2.95 Pa vs. 7.53 ± 2.80 Pa, *p =* 0.104; Table 3). The rWSS_VI-DS_ was different among the three segments of the MCA, with the lowest at the middle segment (0.76 ± 0.35 vs 0.86 ± 0.23 at the proximal segment and 0.99 ± 0.50 at the distal segment, *p =* 0.011, Table 3). With respect to the geometry of the middle segment of the MCA, we found that the WSS of the inner wall was lower than that of the outer wall (6.31 ± 2.08 Pa vs. 8.24 ± 2.46 Pa; *p <* 0.001).

**Table 3:** **Wall shear stress (Pa) of the normal middle cerebral artery**.

Location	WSS_ventral_	WSS_dorsal_	WSS_superior_	WSS_inferior_	WSS _ventral+inferior_	WSS _dorsal+superior_	P	P*
Proximal segment	6.11 ± 2.28	8.08 ± 2.79	7.49 ± 2.63	6.95 ± 2.66	6.53 ± 2.34	7.79 ± 2.60	0.002^†^	<0.001
Mid segment	6.90 ± 2.68	8.84 ± 2.79	8.88 ± 2.64	5.34 ± 1.93	6.41 ± 2.73	8.91 ± 2.76	<0.001^‡^	<0.001
Distal segment	7.00 ± 3.48	7.40 ± 3.26	7.66 ± 3.17	6.19 ± 3.26	6.73 ± 2.95	7.53 ± 2.80	0.131	0.104

P: ventral vs dorsal vs superior vs inferior at 3 different MCA segments; P*: ventral+inferior vs dorsal+superior at 3 different MCA segments.

† ventral vs dorsal, *p =* 0.001; ventral vs inferior, *p =* 0.665; ventral vs superior, *p =* 0.053; dorsal vs inferior, *p =* 0.183; dorsal vs superior, *p =* 1.000; superior vs inferior, *p =* 1.000.

‡ ventral vs dorsal, *p =* 0.001; ventral vs inferior, *p =* 0.015; ventral vs superior, *p =* 0.001; dorsal vs inferior, *p <* 0.001; dorsal vs superior, *p =* 1.000; superior vs inferior, *p <* 0.001.

### 3.2 WSS distribution of atherosclerotic MCAs

**Fig 4:**
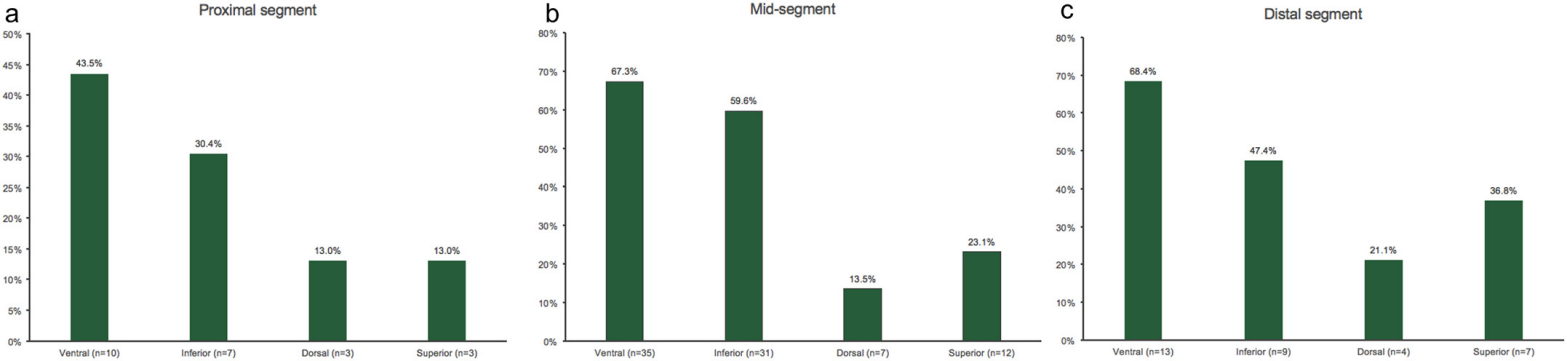
Plaque distribution at different sites of the middle cerebral artery Plaque distribution in (a) proximal segment (b) mid-segment and (c) distal segment of the middle cerebral artery was presented. More plaques formed at the ventral and inferior walls than at the dorsal and superior walls regardless of the middle cerebral artery segments where they were located.

Of the 94 plaques from 43 patients, 52 plaques were located at the middle segment of the MCA, 23 plaques were located at the proximal segment, and 19 plaques were located at the distal segment. Overall, more plaques formed at the ventral and inferior walls than at the dorsal and superior walls regardless of the segments where they were located (Fig. 4). The WSSs of the plaque-containing walls were lower than those of the normal walls regardless of the location of the plaques (6.09 ± 1.96 Pa vs. 7.48 ± 2.39 Pa at the proximal segment, *p =* 0.001; 6.05 ± 2.81 Pa vs. 9.35 ± 3.84 Pa at the middle segment, *p <* 0.001; 5.34 ± 2.70 Pa vs. 9.33 ± 4.41 Pa at the distal segment, *p <* 0.001).

In the proximal and distal segments, 42 plaques were partially located at the bifurcation, leading to difficulties in determining the geometry of the blood vessels in terms of merely curvature. Of the remaining 52 plaques at the middle segment of the MCA, 49 plaques were located at the inner walls of the curve. The WSSs of the plaque-containing inner walls (5.90 ± 2.45 Pa) was much lower than that of the respective normal outer walls (9.52 ± 4.07 Pa; *p <* 0.001).

### 3.3 Difference in WSS ratio between normal and atherosclerotic MCAs

The rWSS_VI-SD_ of the atherosclerotic MCAs was lower than that of the normal MCAs (0.74 ± 0.21 vs 0.87 ± 0.39, *p =* 0.002). Similarly, the rWSS_inner-outer_ of the atherosclerotic MCAs was lower than that of the normal MCAs (0.65 ± 0.17 vs 0.81 ± 0.29, *p =* 0.002).

## 4 Discussion

In this study, we investigated the relationship between WSS and MCA atherosclerosis using a three-dimensional reconstructed WSS model by the CFD technique. In normal MCAs, we observed that the ventral-inferior and inner walls of the middle segment of the M1 curves had lower WSSs. Similar low WSS regions were observed where the most plaques developed in atherosclerotic MCAs. The rWSS_VI-DS_ and the rWSS_inner-outer_ were lower in atherosclerotic MCAs than in normal MCAs.

There have been several studies on models of coronary arteries [17,18], carotid bifurcations [19], and distal abdominal aortas [20], which have demonstrated that low WSS regions were correlated to the localization of atherosclerosis. In regard to pathophysiology, low WSS can enhance the expression of inflammatory adhesion molecules, increase endothelial cell turnover, and decrease the number of antithrombotic factors in arteries, thus initiating plaque formation [21], [22], [23], [24]. In our study, the data lent support to the role of WSS in intracranial atherosclerosis. The finding that most plaques involve the ventral and inferior walls was consistent with previous studies on MCA atherosclerosis [25,26]. Regardless of whether the stenotic MCA atherosclerosis was symptomatic, plaques were more frequently located at the ventral and inferior walls, opposite the orifices of the penetrating arteries [3]. We also observed that the WSS of the plaque-containing inner wall was lower than that of the normal outer wall, which was consistent with our previous observation that the plaques were predominantly distributed at the inner wall of the curved MCA regardless of stenosis degree [12].

The findings of our study are of potential clinical significance. Using the reconstructed WSS model, atherosclerotic-prone regions of the MCA can be identified. Similar “vascular profiling” has been developed by conventional intravascular ultrasound and coronary angiography that can create an accurate and reproducible 3D model of the coronary arteries [27,28]. Such a vascular profile can be individualized and is a promising method for stratifying stroke risk and facilitating prophylactic treatment for intracranial atherosclerotic disease. Furthermore, an in vitro animal study showed a close correlation between low WSS and intracranial in-stent stenosis growth [29]. As a local hemodynamic factor, a normal or relatively high level of WSS maintenance may be helpful in reducing the risk of in-stent stenosis. The evaluation of hemodynamic WSS might be helpful for devising a stent that can create a less thrombogenic microenvironment.

Our study has several limitations. First, the generalization of our results is limited; while the high-grade stenotic atherosclerotic plaque was not included due to the difficulties in establishing the CFD model for this condition. Second, there is no gold standard for direct in vivo WSS estimation. Most previous studies acquired the WSS of the vasculature by combining CFD simulation with digital subtraction angiography [30], MRA or ultrasound [31]. As we did not conduct CTA or 4D FLOW images to verify the accuracy of this technique, further research based on this technique should be carefully performed until the standardization and validation. Third, we applied a constant viscosity in the mathematical model to estimate WSS. Although this is either realistic or subject-specific, recent studies have shown that WSS is not sensitive to a wide range of hematocrits, which is used to estimate viscosity by Einstein's formula, thereby having a limited effect on the estimation of WSS [32,33]. Last, due to the limited sample size, only univariable analysis was performed in this study. Since MCA vessel wall classification and curve orientation may be confounders in the prediction of plaque formation, further studies with larger sample sizes are required to validate the results.

## 5 Conclusion

We built a three-dimensional reconstructed WSS model based on HR-MRI and CFD techniques, which can be used to assess the WSS of MCA. Once validated, it may be a method for stratifying stroke risk and facilitating prophylactic treatment for intracranial atherosclerotic disease.

## Declaration of Competing Interest

The authors declare that they have no conflicts of interest in this work.
